# Prevalence and correlates of night eating syndrome among Chinese nurses: focus on depression and sleep quality

**DOI:** 10.3389/fpubh.2025.1687850

**Published:** 2025-12-03

**Authors:** Shijie Fang, Ruixuan Zhao, Jimin Feng, Hongxu Wang, Dongwen Li

**Affiliations:** 1 North Sichuan Medical College, Nanchong, Sichuan, China; 2 General Hospital of Western Theater Command, Chengdu, Sichuan, China; 3 Chengdu Medical College, Chengdu, Sichuan, China

**Keywords:** night eating syndrome, nurse, depression, sleep quality, cross-sectional study

## Abstract

**Background:**

Nurses are at a heightened risk for Night Eating Syndrome (NES) due to circadian rhythm disruptions from shift work, chronic stress, and irregular eating patterns. While the global prevalence of NES is rising, its status within the large and critical nursing workforce of China remains poorly understood. Specifically, there is a lack of data on the prevalence and unique work-related psychosocial factors associated with NES among Chinese nurses.

**Objective:**

This study aimed to determine the prevalence of NES and identify its key influencing factors among clinical nurses in China.

**Methods:**

A cross-sectional study design was adopted. Using convenience sampling, 311 nursing staff from three Class-A tertiary hospitals in Sichuan Province were recruited as study participants from June to July 2025. Univariate χ^2^ test and Mann–Whitney U test, and multivariate logistic regression analysis to identify the associated factors influencing nurses’ NES.

**Results:**

Among 311 nurses, 14 were diagnosed with NES, yielding a prevalence rate of 4.5%. Statistically significant differences in NES prevalence were observed across departments, alcohol consumption status, night shift assignments, sleep quality, and self-reported depressive symptoms (*p* < 0.05). Multivariate logistic regression analysis further identified the following independent risk factors for NES: working in ICU departments (OR = 14.660, 95% CI: 1.565–137.287, *p* = 0.019), sleep disturbances (OR = 1.339, 95% CI: 1.057–1.696, *p* = 0.016), and depression (OR = 1.083, 95% CI: 1.008–1.164, *p* = 0.030).

**Conclusion:**

This study indicates that, after adjusting for covariates, NES among Chinese nurses is significantly associated with departmental exposure, impaired sleep quality, and worsened depressive symptoms. To address this, hospital administrators should prioritize optimizing night shift scheduling in high-risk departments while integrating sleep management protocols and depression interventions. By preventing NES, these measures can enhance nurses’ physical and mental wellbeing and improve nursing care quality.

## 1 Introduction

Night Eating Syndrome (NES), first described by Stunkard et al. in 1955 (1), is characterized by core features including nocturnal hyperphagia (≥25% daily caloric intake after dinner or ≥2 nocturnal ingestions per week), morning anorexia, and sleep-mood dysregulation. Distinct from binge eating disorder or occasional night eating, this circadian-rhythm-disordered feeding behavior involves dysfunction of the hypothalamic–pituitary–adrenal (HPA) axis, manifesting as aberrantly elevated nocturnal cortisol levels and delayed melatonin secretion (2). In 2013, the Diagnostic and Statistical Manual of Mental Disorders (3) Fifth Edition (DSM-5) formally classified NES under “Other Specified Feeding or Eating Disorders” (OSFED) (4).

In the general population, the prevalence of Night Eating Syndrome (NES) is approximately 1.5%, which can rise to 6–16% among obese populations (5–7). Due to sociocultural differences, the prevalence of NES and its associated factors vary substantially across countries. Specifically, reported rates include 2.8% among Chinese university students (8), 5.3% in Italy (9), and 4.2% in the United States (10). Furthermore, prevalence in the general community is 0.6% in South Korea (11), 1.3% among young adults aged 18–26 in Switzerland (12), 1.5% in Omani adults aged 20 and above (13), 1.1% in Germans aged 14–85 (14), and 8.8% in community samples aged 21–51 in Israel (15). A consistent pattern emerging from these studies is that although the overall prevalence of NES meeting strict diagnostic criteria is relatively low, the proportion of individuals exhibiting excessive nighttime eating behavior is notably higher.

Shift work is a significant risk factor for circadian rhythm disruption and metabolic disorders, markedly increasing the risk of abnormal eating patterns among workers (16, 17). Research indicates that police officers on night shifts not only experience significantly delayed mealtimes but also have a longer daily eating window compared to their rest days (18). Among Korean workers, shift work is associated with various unhealthy eating behaviors, including frequently skipping breakfast, reduced intake of fruits, vegetables, and high-protein foods, although their overall dietary quality shows no significant difference from that of day-shift workers (19). Furthermore, a meta-analysis confirms that night shift work itself increases the risk of metabolic syndrome by 57%, and this risk escalates with longer exposure to night shifts (20). Overall, shift workers commonly face issues such as irregular eating habits, insufficient nutrient intake, and reliance on convenient foods (16).

The nursing profession, in particular, faces heightened risks due to the combination of night shift work and specific occupational stressors (21, 22). As core members of the healthcare team, nurses are responsible for patient care, medical procedures, and continuous patient monitoring. Their work environment is characterized by high psychological pressure, emotional labor, and minimal control over rest periods and schedules (23, 24). By the end of 2024, the number of registered nurses in China had exceeded 5 million, a substantial proportion of whom engage in long-term shift work. Existing studies note that nurses are more prone to abnormal eating behaviors, sleep disturbances, and mood fluctuations (25). These issues are attributed not only to the physiological impact of shift work but also to factors such as limited access to healthy food during night shifts, heavy workloads that prevent normal meal breaks, and persistent psychological stress (26). It is noteworthy that NES may be closely linked to depression and sleep quality (27). On one hand, NES patients may overeat at night, disrupting sleep rhythms and causing circadian rhythm disorders, which may lead to depressive moods. On the other hand, depressive moods may exacerbate night eating behavior, creating a vicious cycle (28, 29). Poor sleep quality may also disrupt appetite regulation mechanisms, making individuals more likely to feel hungry and eat at night, thereby increasing the risk of NES (30). However, no studies have specifically investigated the prevalence of NES among nurses in China. Therefore, gaining a deeper understanding of the current prevalence of NES among Chinese nurses, exploring its relationship with demographic factors as well as depression and sleep quality, is of significant practical importance for implementing targeted intervention measures, improving nurses’ physical and mental health, and enhancing the quality of nursing care.

## 2 Materials and methods

### 2.1 Participants

Conducted from June to July 2025, this study recruited 311 nursing staff via convenience sampling at two Class-A tertiary hospitals in Chengdu and one in Panzhihua, Sichuan Province. Inclusion criteria required: (1) active clinical nurses with >1 year of service; (2) valid Nurse Practicing Certificate of the People’s Republic of China; (3) voluntary participation with signed informed consent. Nurses across all shift types (including day shift only, rotating shifts, and night shift) were eligible to participate, as our aim was to investigate the status of NES in the general nursing population and to understand the role of night shift work as a potential influencing factor. Exclusion criteria eliminated interns, trainees, contract nurses, those with <1 year of experience, and participants withdrawing during the study.

### 2.2 Sample size calculation

**Figure 1:**
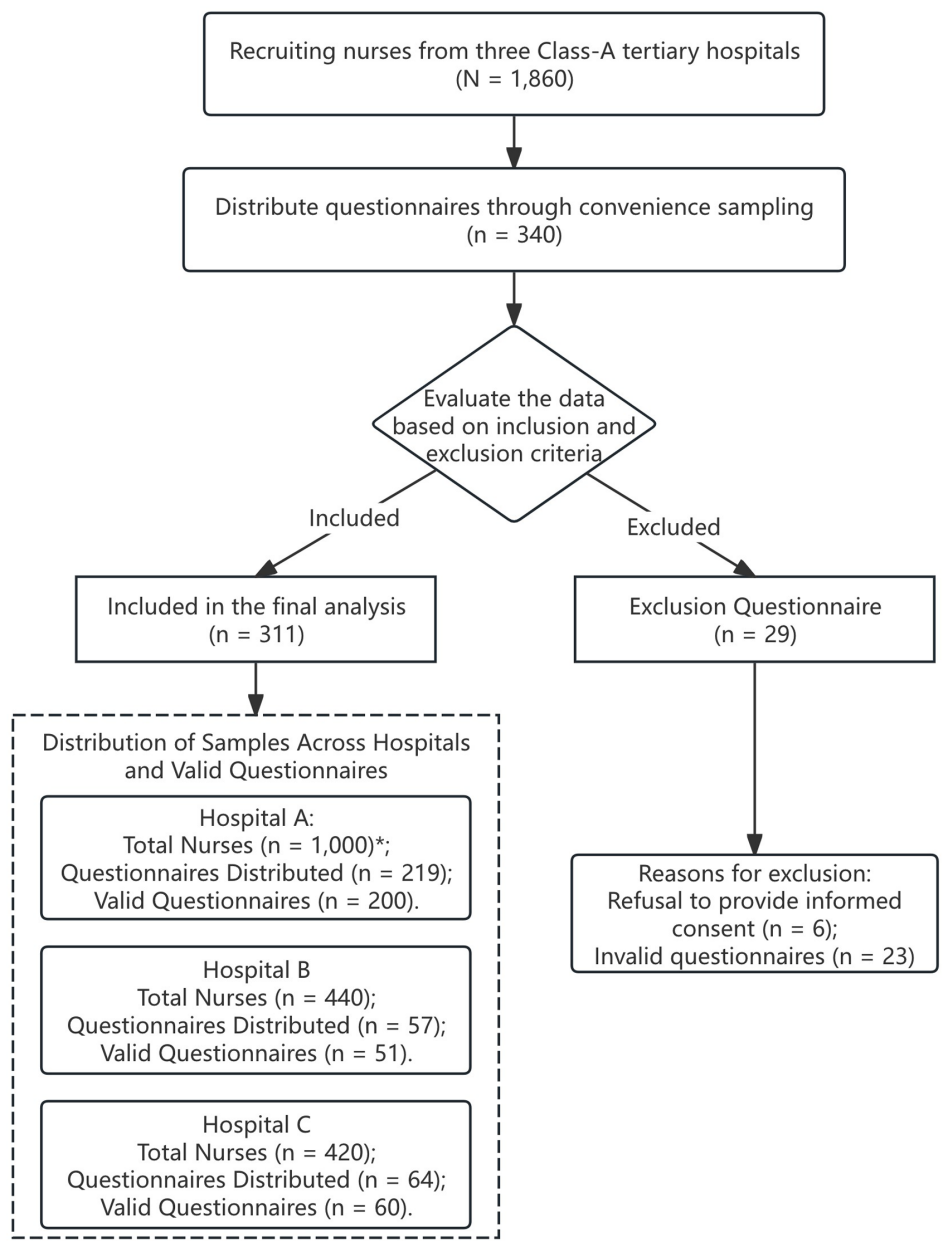
Flow diagram of participant recruitment and sampling. ^*^The total number of nurses at Hospital A is an estimate, as the exact figure has not been publicly disclosed. This data is calculated based on the hospital’s bed capacity.

The sample size was determined using two complementary approaches to ensure both the precision of prevalence estimation and the stability of subsequent regression analyses. First, for the cross-sectional objective of estimating the prevalence of NES, we used the standard formula for a single population proportion (31): $n_{0}=\frac{Z^{2}\times p(1-p)}{d^{2}}$ where *Z* = 1.96 corresponds to a 95% confidence level, *p* = 0.057 represents the anticipated prevalence based on a prior study among nurses (32), and *d* = 0.03 is the margin of error. This calculation yielded an initial sample size of *n*_0_ ≈ 230. After adjusting for a projected 10% rate of non-response or invalid questionnaires, the required sample size became *n* = 230/0.9 ≈ 256. Second, to ensure model stability and validity, sample size calculations were based on the logistic regression standard of 10–20 events per variable (EPV) (33, 34). Fourteen covariates were included (age, gender, ethnicity, department, monthly income, Body Mass Index (BMI), education, marital status, smoking, alcohol use, work hours, night shifts, sleep quality, and depression). To ensure the final analyzable sample contained this number of events after accounting for a projected 10% attrition rate, the initial recruitment target was calculated as follows: 140/0.9 ≈ 156 for the lower end and 280/0.9 ≈ 311 for the upper end. Thus, the target sample size range was 156–311. Our final enrollment of 311 participants successfully met the requirements of both calculation methods, providing sufficient statistical power for constructing the regression model. The participant flow, from recruitment through to the final analytic sample, is detailed in Figure 1. The protocol was approved by the Ethics Committee of Chengdu Medical College (Ethics Number: CMCIR2025NO.021), with all participants providing informed consent.

### 2.3 Measures

#### 2.3.1 Demographic questionnaire

Designed by the investigator, the questionnaire includes the following items: age, gender, ethnicity, department, monthly income, height and weight, highest education level, marital status, smoking and alcohol use, working hours, and night shift assignments. BMI of the participants were obtained from the answers that they provided about their height and weight. Crucially, the questionnaire items explicitly instructed participants to provide values based on their actual situation within the past month, ensuring temporal relevance. BMI was calculated using the formula BMI = weight (kg)/height (m)^2^. Although self-reported data offers practical advantages over field measurements, this method may be subject to social desirability bias (such as underreporting weight) and recall errors, which are more prevalent among individuals with higher BMIs (35, 36). Nevertheless, extensive research indicates that despite systematic reporting errors (e.g., slight overestimation of height and underestimation of weight), self-reported height and weight data show high correlation with measured values (37). The resulting BMI classifications remain valid in large-scale population studies (37, 38). To enhance data quality, biologically plausible ranges were employed as thresholds to exclude outliers. The specific thresholds for height (1.22–2.13 m) and weight (34–227 kg) were defined based on adult anthropometric extremes and practical considerations (e.g., the upper weight limit corresponds to the capacity of common digital scales) (39, 40). All data were programmatically screened against these ranges, with any exceeding values excluded prior to analysis. This method of using predefined ranges for data cleaning is a well-established practice in epidemiological research (39, 40).

#### 2.3.2 Simplified Chinese version of night eating questionnaire

Developed by Allison et al. (41) and translated into Chinese by He et al. (42), comprises 13 items: NE1^*^ Feeling hungry in the morning, NE2 Time of eating in the morning, NE3 Craving food between dinner and bedtime, NE4^*^ Level of control over eating between dinner and bedtime, NE5 Proportion of daily food intake consumed after dinner, NE6 Feeling depressed or low in mood, NE7 Time when feeling low in mood, NE8 Frequency of difficulty falling asleep or maintaining sleep, NE9 Getting up at least once during the night (except to use the restroom), NE10 Craving to eat after waking up in the middle of the night, NE11 Needing to eat to fall asleep after waking up in the middle of the night, NE12 Frequency of getting up to eat after waking up in the middle of the night, NE13^*^ Level of control over eating after waking up in the middle of the night. Items employ a Likert 5-point scale (0 = “Not at all” to 4 = “Extremely”), with asterisked items (*) reverse-scored; total scores range 0–52, where higher scores indicate more severe night eating. A score of ≥25 points suggests the possible presence of NES. The scale demonstrated a Cronbach’s alpha of 0.700 in the original validation (42) and 0.719 in this study.

#### 2.3.3 Pittsburgh Sleep Quality Index

Developed by Buysse (43) at the University of Pittsburgh in 1989 and introduced to China by Liu et al. (44) in 1996, the PSQI is used to assess sleep quality in the general population. A PSQI score greater than seven is used as a reference threshold for sleep quality issues in Chinese adults. The scale includes 19 objective items and four subjective items, totaling seven dimensions: subjective sleep quality, sleep latency, sleep duration, sleep efficiency, sleep disturbances, use of hypnotic medications, and daytime dysfunction. Each dimension is scored 0–3, yielding a global score range of 0–21, with higher scores indicating poorer sleep quality. The scale demonstrated a Cronbach’s alpha of 0.870 in nursing populations (45) and 0.746 in the current study.

#### 2.3.4 Self-Rating Depression Scale

Developed by Zung (46) in 1965, comprises 20 items assessing subjective depressive symptoms using a 4-point Likert scale (1 = “Rarely or none of the time” to 4 = “Most or all of the time”). Ten reverse-scored items (#2,5,6,11,12,14,16,17,18,20) require inverse coding. The total raw score is obtained by summing the scores of all items on the SDS. The standard score is calculated by multiplying the raw score by 1.25 and taking the integer part. According to the Chinese normative standards, SDS scores are interpreted as: 53–62 (mild depression), 63–72 (moderate depression), and ≥73 (severe depression), with a clinical cutoff of ≥53. In this study, the scale demonstrated a Cronbach’s alpha of 0.858.

### 2.4 Data collection methods and quality control

Data collection was conducted via Questionnaire Star, a professional online survey platform. After obtaining authorization from nursing departments of three hospitals, the principal investigator distributed questionnaire links to head nurse WeChat groups. Trained head nurses then forwarded links to departmental groups, explaining the study purpose, significance, and eligibility criteria. An embedded electronic informed consent form on the first page detailed research objectives, procedures, data confidentiality, and voluntary participation—only participants checking “Agree” could proceed. Platform skip-logic functions automated question routing (e.g., if “0” selected on SC-NEQ item nine, items 10–13 were skipped and systematically scored 0 to prevent missing data and maintain scoring consistency). Quality controls included: (1) Device/IP restrictions preventing duplicate submissions; (2) Mandatory responses for non-skipped items; (3) Automated time-tracking to flag invalid responses. Exclusion criteria: responses completed in less than 2 min or those with obvious patterns or illogical answers. A total of 340 questionnaires were collected. After excluding 23 questionnaires due to consecutive identical responses and six due to refusal to participate in the informed consent process, the final analytical sample consisted of 311 nurses (effective response rate: 91.5%). To assess potential non-response bias, *t*-tests and chi-square tests were used to compare the demographic characteristics (e.g., age, gender, ethnicity, department, education level, marital status, monthly income, smoking, alcohol consumption, work duration, shift work, and BMI) between the 23 excluded individuals and the final sample. A significant difference was observed in the age distribution between the two groups (*p* = 0.003), indicating that younger nurses were more likely to be excluded due to consecutive identical responses. However, no significant differences were found in other key characteristics, such as department, alcohol consumption, work duration, shift work, and education level (all *p* > 0.05). Most critically, subsequent univariate analysis revealed that age was not a significant influencing factor for NES.

### 2.5 Statistical methods

Statistical analyses were performed using IBM SPSS version 27.0. Normally distributed continuous data are presented as mean ± standard deviation, analyzed by independent samples *t*-tests; non-normally distributed data are expressed as median and interquartile range, compared via Mann–Whitney U tests. Categorical variables are reported as frequencies and percentages, with group differences assessed by chi-square or Fisher’s exact tests (when expected cell frequencies <5). Pearson correlation analyzed associations between night eating severity (SC-NEQ total score) and sleep quality and depression. Binary logistic regression analysis was used to explore the risk factors for NES, with variables with *p* < 0.1 in univariate analysis included in multivariate modeling. All statistical analyses were performed using two-sided tests, with *p* < 0.05 indicating statistical significance. In order to evaluate the robustness and goodness-of-fit of the model, we performed sensitivity analysis and model checking. Sensitivity analysis was conducted by applying different variable selection methods (Forward and Backward Likelihood Ratio). Model fit was assessed using the Hosmer-Lemeshow test, which indicated excellent calibration between predicted probabilities and observed outcomes (χ^2^ = 3.47, df = 8, *p* = 0.90). Scores from the SDS and the PSQI were treated as continuous variables in all correlational and regression analyses to preserve statistical power.

## 3 Results

### 3.1 General characteristics of study participants

This study recruited 311 nurses, with 97.7% being female. The largest proportion (*n* = 183, 58.8%) were aged 31–40 years, and 97.1% were Han Chinese. Most participants (82.6%) held bachelor’s degrees, while nearly half (50.2%) worked in Internal Medicine departments. Monthly income distribution showed 62.7% (*n* = 195) earned ¥5,000–8,000. Marital status included: unmarried (*n* = 61), married (*n* = 237), divorced (*n* = 12), and widowed (*n* = 1). Non-smokers comprised 97.1% (*n* = 302) and non-drinkers 86.2% (*n* = 268). The predominant work shift duration was 8–10 h per day (*n* = 279, 89.7%), with 70.4% (*n* = 219) working night shifts weekly. Participants’ mean BMI was 21.85 (SD = 3.2; range:15.63–34.60). According to the classification outlined in the People’s Republic of China Health Industry Standard “Adult Weight Classification,” participants were categorized as follows: underweight (BMI < 18.5 kg/m^2^), normal (18.5–23.9 kg/m^2^), overweight (24–27.9 kg/m^2^), and obese (≥28 kg/m^2^) (47). Detailed data are presented in Table 1.

**Table 1:** Demographic details of study participants (*n* = 311).

Variables	Category	Frequency/statistical value	Percentage
Gender	Male	7	2.3%
	Female	304	97.7%
Age, years	≤25	11	3.5%
	26 ~ 30	56	18.0%
	31 ~ 40	183	58.8%
	≥41	61	19.6%
Ethnicity	Han	302	97.1%
	Minority	9	2.9%
Level of education	Secondary specialized education	1	0.3%
	Junior college	52	16.7%
	Bachelor’s degree	257	82.6%
	Master’s or above	1	0.3%
Department	Internal medicine	156	50.2%
	Surgery	60	19.3%
	ICU	12	3.9%
	Other departments	83	26.7%
Monthly income, CNY	<5,000	40	12.9%
	5,000 ~ 8,000	195	62.7%
	8,001 ~ 10,000	51	16.4%
	>10,000	25	0.6%
Marital status	Unmarried	61	19.6%
	Married	237	76.2%
	Divorced	12	3.9%
	Widowed	1	0.3%
Smoking	Yes	9	2.9%
	No	302	97.1%
Alcohol consumption	Yes	43	13.8%
	No	268	86.2%
Daily working hours	<8	17	5.5%
	8 ~ 10	279	89.7%
	>10	15	4.8%
Shift work	Yes	219	70.4%
	No	92	29.6%
BMI	Underweight/normal	247	79.4%
	Overweight/obese	64	20.6%
BMI, kg/m^2*^	M(P_25_,P_75_)	21.48 (20.03, 23.44)	–
	Mean ± SD	21.85 ± 3.20	–

*Since the Shapiro–Wilk test rejected the assumption of normality (W = 0.905, *p* < 0.001). However, sensitivity analysis revealed a 5% trimmed mean of 21.76, differing by <1.5% from the median, indicating that the mean retains reference value. Thus, mean ± SD is additionally reported to facilitate comparisons with prior studies.

### 3.2 Prevalence of NES

Among 311 nursing staff, the mean SC-NEQ score was 13.39, with a maximum score of 33. Fourteen nurses met the diagnostic criteria for NES (SC-NEQ ≥ 25), yielding a prevalence rate of 4.5%.

### 3.3 Nurses’ sleep quality, depression, and night eating behavior scores

The scores for nurses’ sleep quality, depression, and night eating behavior are presented in Table 2. The data are expressed as median and interquartile range.

**Table 2:** Nurses’ sleep quality, depression, and night eating behavior scores, M (P_25_, P_75_).

Project	Total score	Average score per item
SC-NEQ total scores	13.00 (10.00, 16.00)	1.00 (0.77, 1.23)
Morning anorexia	3.00 (2.00, 3.00)	1.50 (1.00, 1.50)
Evening hyperphagia	4.00 (3.00, 5.00)	1.33 (1.00, 1.67)
Mood/sleep	5.00 (2.00, 6.00)	1.67 (0.67, 2.00)
Nocturnal ingestions	1.00 (0.00, 3.00)	0.20 (0.00, 0.60)
Sleep quality	7.00 (5.00, 9.00)	1.00 (0.71, 1.29)
Depression	46.25 (38.75, 55.00)	2.31 (1.94, 2.75)

### 3.4 Correlation analysis between nurses’ sleep quality, depression, and night eating

Spearman correlation analysis revealed significant positive correlations between sleep quality and both SC-NEQ total scores (*r* = 0.638) and its subscales: evening hyperphagia (*r* = 0.337), mood/sleep (*r* = 0.571), and nocturnal ingestions (*r* = 0.462) (all *p* < 0.01). Similarly, depression correlated positively with SC-NEQ total scores (*r* = 0.489) and its subscales: evening hyperphagia (*r* = 0.281), mood/sleep (*r* = 0.467), and nocturnal ingestions (*r* = 0.319) (all *p* < 0.01). Furthermore, a significant negative correlation was observed between the morning anorexia and nocturnal ingestions subscales (*r* = 0.204, *p* < 0.01). Detailed results are shown in Table 3.

**Table 3:** Correlations between sleep quality, depression, and night eating (*n* = 311).

Variable	SC-NEQ total scores	Morning anorexia	Evening hyperphagia	Mood/ sleep	Nocturnal ingestions	Sleep quality	Depression
SC-NEQ total scores	1						
Morning anorexia	0.115^*^	1					
Evening hyperphagia	0.644^**^	0.053	1				
Mood/sleep	0.719^**^	−0.084	0.235^**^	1			
Nocturnal ingestions	0.726^**^	−0.204^**^	0.260^**^	0.319^**^	1		
Sleep quality	0.638^**^	−0.028	0.337^**^	0.571^**^	0.462^**^	1	
Depression	0.489^**^	−0.042	0.281^**^	0.467^**^	0.319^**^	0.561^**^	1

**indicates *P* < 0.01, significant correlation; * indicates *p* < 0.05, significant correlation.

### 3.5 Univariate analysis of factors influencing nurses’ NES

Based on the presence or absence of NES, 311 nurses were divided into a group with NES (*n* = 14) and a group without NES (*n* = 297). The results showed that there were statistically significant differences between the two groups in terms of ICU department (OR = 25.500, 95% CI: 4.862–133.731), alcohol consumption (OR = 5.435, 95% CI: 1.786–16.667), weekly night shifts, poor sleep quality (OR = 1.499, 95% CI: 1.264–1.777), and depression status (OR = 1.113, 95% CI: 1.054–1.175) (all *p* < 0.05). Detailed results for all variables are presented in Table 4.

**Table 4:** Univariate analysis of factors influencing nurses’ NES (*n* = 311).

Variables	Category	Presence or absence of NES		χ^2^/Z	df	OR	95% CI	*p*
		No (*N* = 297)	Yes (*N* = 14)					
Gender	Male	7 (2.4%)	0 (0.0%)	–	1	–	–	1.000^c^
	Female	290 (97.6%)	14 (100.0%)					
Age, years	≤25	11 (3.7%)	0 (0.0%)	4.210^a^	3	–	–	0.233^c^
	26 ~ 30	53 (17.8%)	3 (21.4%)					
	31 ~ 40	172 (57.9%)	11 (78.6%)					
	≥41	61 (20.5%)	0 (0.0%)					
Ethnicity	Han	289 (97.3%)	13 (92.9%)	–	1	1.000	Ref	0.343^c^
	Minority	8 (2.7%)	1 (7.1%)			2.779	[0.323, 23.900]	
Level of education	Secondary specialized education	1 (0.3%)	0 (0.0%)	2.842^a^	3	–	–	1.000^c^
	Junior college	50 (16.8%)	2 (14.3%)					
	Bachelor’s degree	245 (82.5%)	12 (85.7%)					
	Master’s or above	1 (0.3%)	0 (0.0%)					
Department	Internal medicine	153 (51.5%)	3 (21.4%)	19.975^a^	3	1.000	Ref	0.000^c^
	Surgery	54 (18.2%)	6 (42.9%)			5.667	[1.369, 23.448]	
	ICU	8 (2.7%)	4 (28.6%)			25.500	[4.862, 133.731]	
	Other departments	82 (27.6%)	1 (7.1%)			0.622	[0.064, 6.075]	
Monthly income, CNY	<5,000	38 (12.8%)	2 (14.3%)	0.890^a^	3	–	–	0.877^c^
	5,000 ~ 8,000	185 (62.3%)	10 (71.4%)					
	8,000 ~ 10,000	49 (16.5%)	2 (14.3%)					
	>10,000	25 (8.4%)	0 (0.0%)					
Marital status	Unmarried	57 (19.2%)	4 (28.6%)	2.243^a^	3	–	–	0.726^c^
	Married	227 (76.4%)	10 (71.4%)					
	Divorced	12 (4.0%)	0 (0.0%)					
	Widowed	1 (0.3%)	0 (0.0%)					
Smoking	Yes	9 (3.0%)	0 (0.0%)	–	1	–	–	1.000^c^
	No	288 (97.0%)	14 (100.0%)					
Alcohol consumption	Yes	36 (12.1%)	6 (42.9%)	8.342^a^	1	5.435	[1.786, 16.667]	0.004
	No	261 (87.9%)	8 (57.1%)			1.000	Ref	
Daily working hours	<8	17 (5.7%)	0 (0.0%)	0.767^a^	2	–	–	0.600^c^
	8 ~ 10	266 (89.6%)	13 (92.9%)					
	>10	14 (4.7%)	1 (7.1%)					
Shift work	Yes	205 (69.0%)	14 (100.0%)	4.761^a^	1	–	–	0.029
	No	92 (31.0%)	0 (0.0%)					
BMI	Underweight/normal	239 (80.5%)	10 (71.4%)	0.236^a^	1	1.000	Ref	0.627
	Overweight/obese	58 (19.5%)	4 (28.6%)			1.648	[0.499, 5.442]	
BMI		21.48 (19.98,23.39)	21.62 (20.93,24.59)	−1.262^b^	–	1.132	[0.945, 1.357]	0.207
Sleep quality		6.00 (5.00,9.00)	11.00 (10.75,12.50)	−5.106^b^	–	1.499	[1.264, 1.777]	0.000
Depression		46.25 (37.50,53.75)	57.50 (52.50,66.25)	−4.170^b^	–	1.113	[1.054, 1.175]	0.000

^a^ denotes the χ^2^ value. ^b^ denotes the Z value. ^c^ denotes Fisher’s exact tests, Ref denotes Reference group and -denotes not calculable due to zero cells.

### 3.6 Logistic regression analysis of factors influencing nurses’ NES

With the presence or absence of NES as the dependent variable Y (0 = no NES, 1 = NES), all factors showing statistical significance (*p* < 0.1) in the univariate analysis, along with age as suggested, were included as independent variables in a multivariable logistic regression model. The assignment of independent variables is shown in Table 5. The results demonstrated that working in the ICU department, poorer sleep quality, and higher depression scores were independent factors significantly associated with the occurrence of NES among nurses (all *p* < 0.05), after adjusting for other variables including age. For detailed results, please refer to Table 6.

**Table 5:** Independent variable values.

Variable	Assignment
Age	≤25 = 1, 26 ~ 30 = 2, 31 ~ 40 = 3, ≥41 = 4
Department	Internal medicine = 1, Surgery = 2, ICU = 3, Other departments = 4
Alcohol consumption	No = 1, Yes = 2
Shift work	No = 1, Yes = 2
Sleep quality	original entry
Depression	original entry

**Table 6:** Logistic regression analysis of factors influencing nurses’ NES.

Variable	B	SE	Wald χ^2^	df	OR	95% CI	*p*
Internal medicine (reference group)			8.561	3			0.036
Surgery	1.380	0.855	2.606	1	3.976	[0.744, 21.244]	0.106
ICU	2.685	1.141	5.535	1	14.660	[1.565, 137.287]	0.019
Other departments	−0.829	1.275	0.423	1	0.437	[0.036, 5.314]	0.516
Sleep quality	0.292	0.121	5.858	1	1.339	[1.057, 1.696]	0.016
Depression	0.080	0.037	4.724	1	1.083	[1.008, 1.164]	0.030

The multivariate analysis (Table 6) revealed substantial variation in the strength of association between the independent variables and NES. The most pronounced risk factor was working in the ICU department, where nurses had an OR of 14.660 (95% CI: 1.565–137.287) compared to those in internal medicine. This indicates that, after controlling for other factors, ICU nurses had nearly 15 times the odds of developing NES. For continuous variables, the OR represents the change in odds per one-unit increase. Thus, for each point increase in the sleep quality score (indicating worse sleep), the odds of NES increased by 33.9% (OR = 1.339, 95% CI: 1.057–1.696). Similarly, each point increase in the depression score was associated with an 8.3% increase in the odds of NES (OR = 1.083, 95% CI: 1.008–1.164). While working in the surgical department appeared to increase the odds of NES (OR = 3.976), this association was not statistically significant (*p* = 0.106), suggesting the need for caution in its interpretation and further investigation with larger sample sizes.

Sensitivity analysis revealed that poor sleep quality (PSQI score), depressive symptoms (SDS score), and working in the ICU department remained significant predictors of NES across different variable selection strategies (all *p* < 0.05), indicating the robustness of our findings. The Hosmer-Lemeshow test showed good model fit (χ^2^ = 3.47, df = 8, *p* = 0.90).

## 4 Discussion

This study is the first to report the epidemiological characteristics and influencing factors of NES among Chinese nurses. Among 311 nurses, 14 had a SC-NEQ score ≥25, resulting in a prevalence rate of 4.5%. This prevalence, established using a stringent and specific cutoff, provides a robust baseline estimate for the Chinese nursing population. The SC-NEQ screening tool prioritizes specificity, and our figure thus represents a conservative identification of probable cases compared to clinical interviews. Furthermore, the demographic profile of our sample may also reflect the characteristics of the active nursing workforce in China. The clinical and professional significance of this finding is underscored by its position within the broader epidemiological context. While marginally lower than the 5.7% reported among Korean nurse (32), it is significantly higher than the 2.8% prevalence rate among the general college student population in China (8), indicating that nurses exhibit a certain degree of night eating behavior. This may be due to the unique nature of nursing work, which requires shift work. Shift work may alter nurses’ dietary habits, with some nurses adjusting their meal times and frequencies to cope with reduced sleep duration caused by shift work. During night shifts, they may choose snacks to alleviate nighttime fatigue and hunger, highlighting the unique risks associated with the nursing profession (48). Such eating behaviors that contradict circadian rhythms can severely disrupt the body’s normal metabolic rhythm. Long-term night eating may lead to energy metabolism disorders, increasing the risk of obesity, type two diabetes, digestive system diseases, and cardiovascular diseases (49). The incidence rate of NES among nurses is similar to that among Italian college students (5.3%) (9) and American college students (4.2%) (10), further indicating that nurses, like college students in high-pressure, irregular schedules, are a high-risk group for NES. Notably, over 80% of nurses in this study reported cravings for food between dinner and bedtime. The high prevalence of this symptom suggests that the potential NES risk population may far exceed the diagnosed proportion, necessitating early identification and intervention.

An interesting finding in this study is the significant negative correlation between the Morning Anorexia Scale and the Nocturnal Ingestions Scale, which warrants further exploration. First, this result should be interpreted with caution, as multiple studies have indicated that the psychometric properties of the “morning anorexia” subscale within the NEQ itself—such as internal consistency reliability—may be suboptimal (41, 42, 50, 51). Consistent with our findings, prior studies have reported minimal or weak correlations between the morning anorexia subscale and other subscales or the total score (42, 50). Consequently, the validators of the Arabic version of the NEQ recommended prioritizing the SC-NEQ total score over the use of individual subscales with lower reliability (52). This body of evidence suggests that “morning anorexia” may contribute minimally to the core construct of night eating syndrome, functioning more as a descriptive comorbidity than a diagnostic indicator (4, 42, 53). Consequently, the negative correlations observed may partly stem from reliability issues with this subscale as an independent measurement tool. Behaviorally, this finding may also reflect a specific pattern among shift nurses: nighttime eating disrupts circadian rhythms and hormone secretion, thereby affecting morning appetite (54, 55). However, given the aforementioned measurement concerns, this hypothesis requires validation through future studies employing more stable and effective morning anorexia measurement tools.

The results of this study indicate that nurses in the ICU department have a significantly higher risk of developing NES compared to nurses in other departments. This phenomenon is primarily attributed to the synergistic effects of the following three factors: first, circadian rhythm disruption is the key physiological trigger. Frequent night shifts severely disrupt the biological clock, inhibiting melatonin secretion and disrupting the hormonal balance that regulates appetite. During night shifts, levels of leptin—a hormone that promotes satiety—significantly decrease, while levels of ghrelin—a hormone that stimulates hunger—continue to rise, leading to a strong craving for high-calorie foods (56). Studies (57) have shown that night shift and rotating shift nurses have significantly higher cortisol levels than day shift personnel, and their 24-h secretion rhythm tends to flatten, with this endocrine imbalance directly increasing the likelihood of nighttime eating behavior. Second, the chronic accumulation of occupational stress exacerbates the risk of eating disorders. The high-intensity monitoring tasks and complex comorbid care requirements in the ICU keep nurses in a state of prolonged stress (58). Persistent stress stimulation leads to sustained elevations in cortisol levels, and as cortisol is an effective appetite stimulant, it further reinforces the urge to eat, creating a vicious cycle of “stress-cortisol elevation-increased appetite.” Finally, irregular eating patterns exacerbate the issue. Due to the heavy workload and frequent night shifts in the ICU, nurses often resort to fragmented, irregular meal patterns, and even skip meals. Additionally, the scarcity of healthy food options at night leads to night shift nurses consuming predominantly high-sugar, high-fat, and high-calorie foods (48). Research shows (58) that 75% of shift nurses’ energy intake is concentrated between midnight and early morning. This dietary disruption not only directly disrupts insulin secretion rhythms but also further exacerbates cortisol imbalance, significantly increasing the risk of metabolic syndrome. In summary, the interaction between circadian rhythm disruption, occupational stress, and dietary pattern disorders not only weakens the biological clock’s protective mechanisms for health but also poses multiple threats to nurses’ physical and mental health by disrupting physiological systems such as the gut-brain axis, ultimately leading to an increased incidence of NES among ICU nurses.

The primary function of sleep is to promote the restoration of energy and physical strength, and maintaining a regular sleep schedule is crucial for maintaining physical and mental health. However, due to the nature of their profession, nurses often need to work irregular day and night shifts, which inevitably disrupts their normal circadian rhythms. Compared to day shift nurses, shift nurses are often in a state of high stress and are more prone to sleep disorders (49). The results of this study indicate that nurses with sleep disorders have a significantly increased risk of developing NES, with a strong correlation between the two. Nurses with NES generally have higher total scores on the PSQI, directly reflecting poor sleep quality. Additionally, as the severity of NES increases, the severity of sleep disorders also shows a synchronous upward trend. This conclusion aligns with international research findings. A community-based study in Israel (15) found that NES is significantly associated with sleep disorders such as sleep apnea, sleepwalking, and restless legs syndrome. These symptoms not only disrupt the sleep process but also create a vicious cycle of “poor sleep-nighttime eating.” From a pathophysiological perspective, NES patients often experience delayed sleep onset due to excessive evening eating and frequent nighttime eating, leading to insufficient sleep and reduced sleep quality. Additionally, reduced levels of melatonin and leptin in plasma further disrupt sleep rhythms while exacerbating nighttime eating impulses, creating a bidirectional influence (1, 15, 59). Studies specifically targeting nursing populations also support these findings. A survey of pediatric intensive care unit (PICU) nursing staff revealed that sleep disorders are prevalent in the nursing profession due to night shift work, severely disrupting personal life and social functioning; Domestic studies on intensive care unit (ICU) nurses also indicate that high-intensity shift work, frequent overtime, and stress from emergencies easily lead to sleep deprivation and sleep disorders (49), laying the groundwork for the development of NES. These studies collectively reveal the complex mechanisms of interaction between sleep disorders and NES in the nursing population.

Our study found that 28.9% of nurses reported mild or more severe depressive symptoms, a proportion significantly higher than previous studies. Further analysis using binary logistic regression revealed that, after controlling for confounding factors, nurses with NES had a 1.083-fold higher risk of depressive symptoms compared to those without NES, confirming a positive correlation between NES and depressive symptoms. This conclusion aligns with the results of multiple previous studies. From an international research perspective, a cross-sectional study of 404 Korean nurses indicated that NES patients had a 1.65-fold increased risk of depression (32); in a sample of 1,514 young adults aged 18–26 in Switzerland (12), the NES group reported more pronounced eating disorder pathology and depressive symptoms compared to the healthy control group; Geliebter’s team (60) and clinical observations of patients with major depressive disorder both reported a significant association between NES and the severity of depression (61). In domestic studies, He et al.’s survey of NES among Chinese college students (8) confirmed a significant positive correlation between NES scores and psychological distress through univariate analysis; a large-scale study of 3,278 Chinese college freshmen also validated the significant association between NES and depressive symptoms after adjusting for covariates (62). Additionally, a study of medical students in Saudi Arabia used binary logistic regression analysis to further confirm a significant association between NES and depression among medical students (63), highlighting the complex interplay between mental health and eating behavior. From a pathophysiological perspective, the onset of NES may be associated with an increase in specific serotonin transporters, leading to a reduction in total serotonin levels in the synaptic cleft. It has been widely established in the academic community that reduced serotonin levels in the circulation are closely associated with an increased risk of depressive symptoms. From a physiological mechanism perspective, this provides theoretical support for the association between NES and depressive symptoms (64, 65).

Interestingly, our study results showed that drinking and night shifts were significantly associated with NES in univariate analysis, but after controlling for variables such as sleep, depression, and department, the results of multivariate logistic regression analysis showed that the independent effects of drinking and night shifts disappeared. This finding suggests that night shifts may indirectly trigger NES by worsening sleep and emotional states, rather than being a direct cause of the condition; meanwhile, alcohol consumption may serve as a tool for emotional regulation among nursing staff, rather than an independent causative factor. Additionally, our study did not find statistically significant differences in NES across gender, age, body mass index, monthly income, education level, smoking status, or work hours. However, the relationship between NES and these factors remains a topic of debate in existing research. Previous studies have reported gender differences, with men having a relatively higher incidence of NES than women (8, 64, 66–68). Given that our study focused on a nurse population predominantly composed of women, this inconsistency may be due to differences in the gender composition of the samples used in different studies. Other studies have established a positive correlation between NES and BMI, with individuals experiencing NES often having higher BMI (68, 69). The complexity of this relationship underscores the need for further research to fully elucidate the factors contributing to NES and the variability in its prevalence.

## 5 Limitation

The limitations of this study should be acknowledged. First, the use of convenience sampling from three hospitals in two cities may limit the representativeness of the sample, particularly regarding geographic diversity and hospital level variation, which could affect the generalizability of the findings to nurses in other regions or healthcare settings. Second, although the response rate was high (91.5%), data were collected online via WeChat-based surveys, which may have introduced selection bias by underrepresenting nurses less active on digital platforms. Additionally, among the 23 excluded responses, participants were significantly younger than those included (*p* < 0.05), suggesting that very young nurses might have been more likely to provide incomplete or invalid data—a pattern that warrants attention in future online surveys. Third, the sample was predominantly female (97.7%) and Han Chinese (97.1%), which limits the applicability of the results to male nurses and ethnic minority groups. Fourth, BMI was self-reported rather than measured, which may have introduced reporting bias and misclassification of weight status. Fifth, the diagnosis of NES was based on a self-reported screening tool (SC-NEQ) rather than clinical interviews, which may affect diagnostic accuracy. Sixth, the limited number of NES cases (*n* = 14) resulted in a low EPV ratio in the regression analysis. Although we employed a dual-method approach for sample size justification and used statistical techniques to optimize the model, the EPV heuristic itself is not a precise method as it does not account for effect size, variance, or statistical power. This may lead to overfitting and unstable parameter estimates, and the multivariate results should therefore be considered preliminary. Finally, as a cross-sectional study, this design cannot establish causal relationships between NES and sleep or depressive symptoms. Future studies should employ prospective designs to clarify causal pathways, incorporate stratified sampling across diverse regions and hospital levels, utilize mixed-mode data collection to reduce digital participation bias and enhance engagement among younger nurses, and combine objective measurements (e.g., measured BMI) with semi-structured interviews to improve the validity of NES and anthropometric assessments. Finally, results from multivariate logistic regression should be considered preliminary and exploratory, requiring validation in future large-scale epidemiological studies.

## 6 Conclusion

The results indicate that NES is present among Chinese nurses, with a notably higher prevalence observed among those working in the ICU. Night shifts and alcohol consumption may indirectly influence nurses’ night eating behavior through mediating variables. Additionally, NES is closely associated with higher levels of psychological distress and sleep disorders. Given that this study was conducted among nurses in three hospitals in Sichuan Province, the findings provide valuable preliminary evidence but may not fully represent all Chinese nurses. Therefore, multicenter or national-level studies are necessary to verify the prevalence of NES and its associated factors before implementing large-scale, targeted public health interventions. Based on the associations identified in this study, healthcare institutions may prioritize the prevention and management of NES. For instance, to mitigate circadian rhythm disruption associated with night shifts, exploring scientifically designed scheduling systems may be beneficial (e.g., extending night shift cycles and reducing the frequency of consecutive night shifts). Additionally, creating a supportive environment that may help alleviate work stress could include evaluating the provision of nutritious meal options during night shifts and establishing comfortable rest areas. Future intervention studies are needed to directly test the effectiveness of these specific measures in reducing NES among nurses.

## Data Availability

The raw data supporting the conclusions of this article will be made available by the authors, without undue reservation.
